# "Reviewing Our Series, Three Cases Only Died"

**Published:** 1970-07

**Authors:** Alan B. Raper


### "Reviewing our Series, Three cases only died"

Alan B. Raper

As speech, my title would be quite intelligible; appropriate pauses, and the rise and fall of the voice, would see to that. But written as it is, it supplies some innocent fun. I see the "cases" first—they have pulse charts and electrocardiographic tracings, but no hearts; they have fluid outputs but they do not go to the lavatory; their faces are merely something in the way of a view of the pituitary fossa; in short they are true cases, masses of facts, opinions, unanswered questions, arguments, records; and as such they cannot suffer death. But let us admit that medical jargon allows us to speak of people in this way. Now, I want to know what business had these three people to be "reviewing our series"? For this is what the sentence says they were doing. Alas, it was fatal to them. But take heart, they "only died", so compared with other things they might have done, their deaths were a trivial matter.

Such are the results of putting down one's speech on paper—it may rise up transfigured. My thesis here is the simple one that speech is not writing, and that thoughts expressed in speech need careful consideration before they are turned into writing, with special reference of course to medical writing.

Speech communicates by words, emphasis, tone of voice, and even by gesture and facial expression. It is transient and generally trivial; it abounds in clichés; words are strung together in an order dictated by habit, not strict sense. However, it can be subjected to immediate checking, cross-questioning, and correction, so that the true message may emerge. But writing is final, and being insusceptible of questioning or checking it must be above the need for further explanation. Everything hinges upon the fact that the only access that the reader has to the writer's train of thought lies through those printed words, and they have to convey their meaning by being well chosen, well ordered, properly punctuated, and in accord with grammar and idiom.

The marks of bad or ineffective writing are therefore ambiguity, offence against idiom, and bad grammar. These are commonly combined, but it is quite possible for an unambiguous statement to offend by its clumsiness, or for a grammatically correct sentence to be made incomprehensible by ill-considered punctuation. A musician must not utter false notes; a boxer must abide by the Queensberry Rules; a motorist must respect the Highway Code; for anyone who breaks the rules causes confusion and distress, and must expect at least to be mildly cautioned. So it is with the writer. His business is communication by the written word, and for this there are certain conventions.

Before I consider how medical writing most frequently falls short, I must make it quite clear that I am neither a purist nor a grammarian, still less an infallible writer. Indeed anyone who has written must know that he has but to show his script to a colleague for innumerable faults to be revealed—nor should any medical writer omit this test. And I am concerned only with matters that made the difference between just tolerable and good writing in medical papers. Many of the examples I quote are drawn from printed works, that is, they have undergone the process of editing; there is a lesson here, and if it relates to how much one can trust the editor, it also relates to how much one should ask him to bear.

I make no apology for beginning with that simplest of signs, the comma. After the words themselves, it is chiefly the comma that moulds written sentences, and supplies the eye with the necessary signposts. H. W. Fowler, in his 'Modern English Usage' says "One of the first requisites for the writing of good clean sentences is to have acquired the art of enumeration, that is, of stringing together three or four words or phrases of identical grammatical value without going wrong". It would be very difficult to find a medical article that does not contain a list of items, even if it be only a list of the authors of another article. That list is a convenient starting point for a consideration of the comma. A very common practice is to write "Smith, Brown, Jones and Robinson". Yet this has the effect of setting Jones and Robinson apart, as if they had an association more constant and more intimate than that between Smith and Brown. There may be occasions when it is appropriate to do just this, for example in such a list as "Buster Keaton, Chaplin, Laurel and Hardy". But now notice the effect of a comma; if we write "Buster Keaton, Chaplin, Laurel, and Hardy", the last names at once spring out as those of two separate persons. And surely this is the way in which we think of, say, "Coombs, Mourant, and Race (1945)", as three men granted equality by the final comma before 'and'. This may seem to be a minor quibble, but it contains a principle of great importance to clarity. To invent a somewhat knockabout illustration: "In this kinship were to be found persons with grey, brown, black and blue eyes". Common sense, of course, is the clue. When it gives way to rule of thumb (No comma before 'and'!) as is the practice in some printing houses, folly creeps in, and presents us with such gems as "The German delegate declared the Baltic countries, Finland and Rumania, to be within Germany's sphere of interest". Notice that one does not need special knowledge to correct that sentence; but what if the list had been of abstruse items? How should we interpret a medical writer's intention (and assess his knowledge) if he wrote of his patients as showing "the complications of diabetes, hypertension and cirrhosis?" Unambiguous enumeration is indeed necessary for clarity.

The comma is a signpost to the eye, not a signal to take a breath. Test its function in this sentence: "Britain had gone to war with Spain in 1739 largely over the danger to her West Indian interests and Newcastle, in the hope of retaining Louisburg with its implied threat to French Canada, had postponed the end of the war with France until 1748". The idea of Newcastle—the city or a man — being in danger is vaguely disturbing; next, the question of Louisburg comes up as if in a list of the causes of the war; but the sentence remains inscrutable until we reach, with a jolt, the 'had' after the second comma. This identifies Newcastle as a man, and automatically puts Louisburg in its place in the war strategy. A comma after 'West Indian interests' would not have cured the sentence of its clumsiness (re-writing alone could do that), but it would at least have given a clue earlier. The more involved the sentence, the greater is the importance of correct signposting. Try to extract the meaning—at a first continuous reading—from this sentence—"Whether an embolus remains in the main pulmonary vessels, fragments to impact peripherally in the pulmonary vascular bed or undergoes shrinkage, lysis or organisation depends upon factors such as the structure and size of the embolus, whether further embolisation occurs from the peripheral veins and the fibrinolytic activity of the endothelium in the pulmonary artery and perhaps of the blood". I am sure the writer understood the situation perfectly. You or I could re-punctuate his sentence into clarity — but could we be sure of achieving the original meaning? Alas, we shall never know who laid all the false scents, the writer or the editor. Certainly the lesson is that a sentence shoud never leave our hands until the distribution of the commas is such that none but the most inattentive reader could be led astray, or forced back to pick up lost scents.

Lists commonly appear in medical articles to describe the various steps of some process. In a "Methods" sections one can find these strings of "phrases of identical grammatical value", so that we are still concerned with Fowler's problem of enumeration. I want to draw attention to the way in which verbs and their subjects tend to become wildly scrambled in these lists. A simple example is "The deposit was washed three times, packed, and the supernatant discarded". The 'was' is doing its duty to 'deposit' when it controls 'washed' and 'packed'; but it has no obligation to serve 'supernatant', which is in a separate clause, and needs its own verb, which in this case happens to be 'was'—but a quite different 'was'. Still worse, "The cells were washed, packed, and the supernatant discarded", for now confusion in number is added, if it is hoped that the 'were' can serve 'supernatant'. Examples of this sort can be found in almost any scientific medical paper; often such sentences are long and involved, and one can understand that the writer wants to get the list finished with as few words as possible, so that he grossly overworks his first 'was'. If he must save words he could use the "notes" form: "Wash and pack cells; discard supernatant; re-suspend cells". But if he opts for running prose he must not make the reader dizzy by his antics.

In other contexts, too, it is a common and simple error to mistake the correct subject of a verb; it is often the introduction of a passive construction that lays the trap, sometimes with comical results. We are all guilty of turning "A licence was issued to me" into "I was issued with a licence". Medical writers are more prone to the absurdity of turning "The patient received an injection (or transfuson) of X" into "The patient was injected (or transfused) with X". We say it in speech, but we should not admit in writing that we put our patients into syringes. By similar means, non-medical writers may surprisingly enter our field: "The town was evacuated" says, and means, that the town was made empty, but by confusion of subject there arises the expression "The townsfolk were evacuated", and what was perhaps a precautionary military measure becomes a drastic medical one. In some situations, reasoning from first principles may have to give place to idiom; a woman is delivered of a child, but a child is not delivered, it is borne by a woman, and finally it is born.

An adverbial clause, especially when it begins a sentence (and how common that practice is, as in my title!) very readily confuses our feeling for the subject. "While screening expectant mothers for anaemia, a woman was found who had leukaemia". This asserts that the woman with leukaemia was doing the screening. It is forgotten that the as yet unmentioned subject of 'screening' is really 'we' or 'the author', and the comma should be followed by 'we found'. Examples of this lack of sure-footedness in writing can be found daily.

A false analogy between expressions leads to ambiguities or even nonsense. For example, if X is replaced by Y, then it is correct to say that Y is substituted for X. But 'substitute' is a verb that defeats most of us; we cannot remember that one thing substitutes for another, and so we may say "X was substituted by Y", and leave the reader to guess what happened. Here we use 'by' because of the feeling that we really ought to be using 'replaced by'—and indeed it would be safer to do that. A useful guide with 'substitute' is to think of it as a noun, and then we have no difficulty in think of A (say a footballer) as a substitute for (the injured) B.

False analogy, aided by a desire for elegance, is the basis of an expression often found in "scientific" papers. "1 ml. is placed into a cuvette". In ordinary language we should say 'put into'. But 'put' seems too simple a word for this careful operation, so 'placed' is selected to convey the idea of a motion both solemn and elegant. No thought is then given to changing 'into' at the same time; yet 'placed in' is idiomatically correct, conveying the idea that when a thing is placed it is no longer in motion, and must be in, or on, or under something. 'Into' refers to motion, 'placed' is static. To conjoin them offends the reason. It does not lead to ambiguity, it merely saddens the reader.

From the foregoing examples, it will be seen that when we have to cast about for a preposition to use, we are inclined to decide on the basis of analogy. It is confused thought, or lack of a sure sense of idiom, that yields to the guidance of a false analogy.

But there are some situations in which idiom is not secure; it is in fact changing, and neither the old nor the new is in full possession. 'Different from' and 'different to' are in this state. 'To' puts up a strong fight because it is so easy to say, and it may win in the end, despite the very obvious sense of differing 'from'. Commonsense is less emphatic in the question of 'identical with' (English) and 'identical to' (American). Most people would on reflection choose 'with', in the sense of sharing, or having in common, all characteristics 'with'. But many, in haste, would accept the easier 'to', and derive some assurance from 'equal to' and 'similtar to', although these are not quite fair comparisons as they do not express oneness, but only comparability. Probably the very oneness of 'identical' should discourage the use of any preposition, and "X is identical with Y" is better expressed by "X and Y are identical". The two meanings of 'identity' can cause confusion; here is an example: "The components were found to be $\alpha$ and $\beta$ chains, the identity of which was confirmed by . . ." This sentence says that there was no difference at all between $\alpha$ and $\beta$ chains. But it meant to say that each component had been separately identified, and this could have been achieved by " . . . the identities of which were confirmed by . . ." In fact 'identity' and 'identical' are dangerous words, not only in grammar but also in logic, and in most scientific contexts the true logical conclusion, derived from the facts available, is less one of identity than one expressed by "X is indistinguishable from Y".

Probably the most disfiguring disease that otherwise healthy writing can suffer from is connected with 'due to'. Idiom decrees that one state of affairs, or event, can be 'due to' another thing or event. That is, 'due to' relates, as cause and effect, two nouns or substantival clauses; it is not a conjunction. Hence, "The improvement in his condition was due to the nursing care he received", but never "His condition improved, due to the nursing care . . . " Whenever 'due to' springs to mind as one writes—and it is always doing so—one should at once stop and question it. Does it directly connect cause (substantive) with effect (substantive)? If not, then either the real substantive must be found and inserted ('the improvement', above), or else 'due to' must be replaced by 'because', 'because of', or 'owing to'.

'Due to' demands another paragraph, because it abounds in medical writing, and its bad usage is quite as common as its good. Let us agree (we have no choice) that "Intestinal infections were common, due to the insanitary conditions in which the men worked" is bad grammar. It is also, and of course necessarily, bad thought and bad communication. For though the cause in this sentence (insanitary conditions) is clearly stated, we cannot tell whether the effect is the occurrence of infections, or their commonness. Correct writing will communicate what sequence is intended:—"Intestinal infections, due to the insanitary conditions . . . were common". "Intestinal infections were common because of the insanitary condition . . . " "(The fact) That intestinal infections were common was due to the insanitary conditions . . ." For practical purposes, it can be said that any sentence that begins with "Due to . . . " is doomed. The exceptions are negligible. After such a beginning, the grammar can only be unscrambled by clumsy inversions: "Due to the insanitary conditions in which the men worked was the frequency of intestinal infections", and no one would buy correct grammar at that price. The "Due to . . . " sentence is not only abortive, it sticks out like the familiar sore thumb by reason of its capital D, and that is why I say it disfigures writing.

'Only' is less dangerous, but it is one of the most slippery of words to handle. It has the habit of appearing in the wrong place in a sentence, and of grappling onto a word not intended for it. It has a particularly strong affinity for a following verb. In my title, there is nothing to show whether it should be attached to 'cases' or to 'died'. To free 'died' from its clutches, one has either to place 'only' before 'three cases', or to erect a new barrier such as "three cases only, out of 20 treated, died"'. 'Only' can find a place for itself in many different positions in a given sentence, each time giving rise to a different nuance in the meaning. It is very easy to let it place itself wrongly, especially in the heat of composition. Fortunately there are many occasions when this does matter; the reader unconsciously adopts the right meaning, or perhaps consents not to be too critical. Nevertheless, the heat of composition over, the writer should carefully scrutinize every 'only' as he re-reads his script. For surprises surely await him, even reversals of his meaning, as in "The drug only seems to be effective when given intravenously".

The word 'data' is commonly and cruelly misused. It is astonishing how often it is used as a noun in the singular. That is simply barbarous, and it is no excuse for the writer to say that what he had in mind was 'this collection of data'. But the word is more subtly insulted. Data are facts of nature or experiment, they are not the concepts that spring from a consideration of these facts. If concepts and deductions are being discussed, with a few facts intermingled, as often happens in the "Discussion" section of a paper, one cannot begin the next sentence with "These data indicate . . . " But it is regularly done. 'Data' is so crisp and useful in its own sphere, that it is a harmful act to widen and diffuse its meaning.

Turning from such gross lapses, we might look at some minor irritations. Some writers almost compulsively begin sentence after sentence with 'thus' when 'for example' is meant, or indeed no preliminary noise is needed. Minor mistakes with 'thus' are not particularly irritating; they only become so when frequently repeated. Even 'the' has its dangers. "This disease is common in the African" sounds unexceptionable in English ears. But consider "Obesity is common in the stockbroker". We recognize at once that to use 'the' with the singular noun reduces all stockbrokers (or Africans) to an anonymity which, applied to persons, can be insulting. The simple plural (stockbrokers, Africans) removes the slur that a sensitive member of either group might have felt, for his group is now recognized to consist of persons, not a mass of humanity that the writer disdains to see as individuals. The criterion here is neither grammar nor logic, it is common humanity; compare, for example, the wholly acceptable "Blessed are the pure in heart", which defines a sub-group by individual assessment.

This brings me back to 'cases' as a synonym for patients or persons. It may be common, almost universal, medical jargon, though this alone does not condemn it, at any rate if we can be sure we are addressing it to medical men only. But it de-personalizes a human being. It also robs the writer of the flexibility that the correctly used word can give. How much more pleasing it is to see it takes its proper place in a sentence such as "Nausea was experienced by 15 subjects, but in most cases it was transitory".

I have suggested that to a medical audience medical jargon makes sense—so why not use it? Well enough, if we are speaking face to face. But a writer's audience is unknown to him; it may not be wholly medical, or it may be the medical audience of a century hence, responsive to a different jargon. True, "He had a positive Babinski" cannot mislead, for if it is not understood it can only confound the reader. But 'case' is a word of everyday usage, and it is unfair to appropriate it for jargon use, especially when that usage robs the subject of his humanity.

Another jargon word is 'parameter'. It has had a long and respectable use in mathematics. But it was no sooner discovered by medical writers in search of elegance and erudition than it underwent (as we should say in pathology) de-differentation, which suggests (in the same context) that it became malignant. It means yard-stick, or standard. Anaemia is assessed by measuring the haemoglobin level in the blood. The haemoglobin level—as a concept—is thus a parameter of anaemia. But the actual haemoglobin level of a patient is not a parameter, it is a measurement, a specific instance, to be judged by reference to the proper parameter. So, in commenting on four graphs, each showing the distribution of a set of observations, it is otiose to say "It is apparent from the distribution of these parameters . . ." Or, in another instance, "Appreciable differences existed in these three parameters" among the subjects, meaning of course "in these measurements", or "in the mean values for X, Y, and Z". The special, useful, meaning of the word is killed by such usage; it is as if we were to obliterate 'gas' and 'meter' by making 'gasmeter' serve for both. I do not wish to decry the temporary allocation of a word to some specialized meaning; words used metaphorically can be illuminating, and are indeed the hall-mark of sensitive writing ("How sweet the moonlight *sleeps* upon this bank"). So I cannot object to the old seizure of 'murmur' by cardiologists, or even the newer vogue-words 'pool' and 'compartment', for none of these usages robs the languages of life and flexibility.

This leads to a final reflection. A living language is constantly changing, and it would be foolish to resist change just because it is change. Speech leads in this, and writing tends to fix the changes. The writer should, if he values his language, consider whether the changes he commends by his use of them are for the better. In changing, words may become more, or less, specialized in meaning; or one word may be retained for a special meaning while its near relation moves off to meet a new and slightly different demand. This is differentiation, and as long as our mental concepts expand, it is a necessary and valuable process. But loss of differentiation, or degradation in meaning ('fantastic'), is almost always a true loss to language and thought. Idioms change too, but more slowly; again, speech leads and writing follows, and this is reasonable because, as I said before, writing must be unambiguous, and it cannot therefore adopt changes until they are almost universal in speech. English is more receptive of change than, say, French. But the writer of English should always consider whether he is improving or degrading the language. There are text-books on this subject, and the medical writer should find them just as worthy of study as medical text-books. But craftsmen best become masters of their art by contemplation of the work of earlier masters, and by rejection in their own work of anything falling short of the best they can do.

The examples and the principles that I have discussed are simply those that have struck me as the most obtrusive while reading medical articles during the last year or so. Many more could be collected. And no doubt different readers would have been sensitive to different mistakes, even to the extent of disagreeing with some of my conclusions. Indeed I hope this is so, for the main purpose of my writing is not to condemn, but to foster sensitivity to the appearance and manifest content of medical literature. But disagree in detail how you will, I hope there will be full agreement on the result to be achieved — that is, forceful, illuminating, sensitive, unambiguous communication.

